# Slim by Design: Serving Healthy Foods First in Buffet Lines Improves Overall Meal Selection

**DOI:** 10.1371/journal.pone.0077055

**Published:** 2013-10-23

**Authors:** Brian Wansink, Andrew S. Hanks

**Affiliations:** 1 Charles S. Dyson School of Applied Economics and Management, Cornell University, Ithaca, New York, United States of America; 2 Department of Applied Economics and Management at Cornell University, Ithaca, New York, United States of America; University of Chicago, United States of America

## Abstract

**Objective:**

Each day, tens of millions of restaurant goers, conference attendees, college students, military personnel, and school children serve themselves at buffets – many being all-you-can-eat buffets. Knowing how the food order at a buffet triggers what a person selects could be useful in guiding diners to make healthier selections.

**Method:**

The breakfast food selections of 124 health conference attendees were tallied at two separate seven-item buffet lines (which included cheesy eggs, potatoes, bacon, cinnamon rolls, low-fat granola, low-fat yogurt, and fruit). The food order between the two lines was reversed (least healthy to most healthy, and vise-versa). Participants were randomly assigned to choose their meal from one line or the other, and researchers recorded what participants selected.

**Results:**

With buffet foods, the first ones seen are the ones most selected. Over 75% of diners selected the first food they saw, and the first three foods a person encountered in the buffet comprised 66% of all the foods they took. Serving the less healthy foods first led diners to take 31% more total food items (p<0.001). Indeed, diners in this line more frequently chose less healthy foods in combinations, such as cheesy eggs and bacon (r = 0.47; p<0.001) or cheesy eggs and fried potatoes (r = 0.37; p<0.001). This co-selection of healthier foods was less common.

**Conclusions:**

Three words summarize these results: First foods most. What ends up on a buffet diner’s plate is dramatically determined by the presentation order of food. Rearranging food order from healthiest to least healthy can nudge unknowing or even resistant diners toward a healthier meal, helping make them slim by design. Health-conscious diners, can proactively start at the healthier end of the line, and this same basic principle of “first foods most” may be relevant in other contexts – such as when serving or passing food at family dinners.

## Introduction

Each day millions of people pick up a plate at the beginning of a buffet line. Many of these people will not even directly pay for the food they take because they are not at a buffet restaurant. Instead, they are conference goers lining up at a lunch buffet, travelers eating a complimentary hotel breakfast, college students on a meal plan, military personnel eating on base, or one of the 31 million K-12 graders eating a USDA-reimbursable school lunch.

Given the growing prevalence of obesity ([1], [2], [3]), a number of recent studies have examined how to redesign cafeterias and buffets to guide or nudge diners to eat less and eat better. Many of these studies have utilized principles from behavioral economics and have generally focused on at least one aspect of the *CAN*-method – making the healthier foods more *C*onvenient, *A*ttractive, or *N*ormative ([4], [5]). Convenience is achieved in many ways such as by placing more healthful foods near, or next to, the cash register, or introducing a grab-and-go line ([6], [7], [8]). Attractiveness can be achieved by giving healthy foods more enticing names, placing them in a nice bowl, or softening the music or lighting ([9], [10]). Healthy foods can be made more normative by making them – or a reduced portion size – appear more normal, common, or cool ([11]).

Most of these studies generally focus on one or two foods (such as a fruit or vegetable) in isolation from other food items. What is often overlooked is that each food that is taken is either *substituted* for another food or taken *in addition* to other foods on the serving line. In other words, each food taken may partly determine what other foods a person selects. In this way, the first food a person selects could trigger subsequent selections of complementary foods [12].

Imagine two arrangements for a buffet. Foods in the one arrangement are ordered such that the first foods one sees are the healthier, relatively less caloric foods (such as fruit, low-fat yogurt, and low-fat granola), but foods in the other arrangement are ordered such that the first foods one sees are the less healthy and relatively more caloric foods (such as cheesy eggs, fried potatoes, and bacon). In the study presented in this paper, two breakfast buffet lines were arranged in this way in order to answer the following three research questions: 1) Are diners more likely to take the first foods they see? 2) Does taking the first item trigger subsequent choices? and 3) Are there differences in the total number of foods chosen between the two lines? Answers to these questions have key implications for how a wide range of buffets, cafeterias, mess halls, and school lunch lines are arranged. For health-conscious diners, it could also influence which end of a buffet line they choose to proactively approach first.

## Methods

To examine how the food order in buffet lines can influence food choice, we observed the foods that conference attendees served themselves from a full breakfast buffet line. These diners were Human Resource managers attending a conference on behavior change and health. Two identical and separate serving lines were set up approximately 54 feet from each other. While there was no difference in the type or amount of food in both lines, food order was reversed between the two lines. The basic order was determined by a registered dietitian and was partially based on the caloric and nutrient density of the foods. On one line, cheesy eggs were served first, followed by fried potatoes, bacon, cinnamon rolls, low-fat granola, low-fat yogurt, and fruit. Food on the other line was ordered opposite to that on the first line: fruit, low-fat yogurt, low-fat granola, cinnamon rolls, bacon, fried potatoes, and cheesy eggs.

As conference diners entered the main door to receive their breakfast ticket, they were randomly assigned to one of the two buffet tables and then escorted to their respective table in an alternating sequence. At this time diners were told because of scheduling and logistics they could only make one trip to the buffet. Because some diners arrived in groups of two to four, steps were taken to organically keep them with their group and to escort the entire group to one of the buffet tables, and then to escort the next small group or individual to the other table. In all, 124 individuals passed through the two breakfast buffet lines; 59 passed through the fruit-first line, and 65 passed through the cheesy eggs-first line.

Cornell University’s IRB issued a formal waiver for the need of ethics approval and informed consent, as no identifying information was collected from participants. The researchers had no direct contact with the diners other than escorting them to a buffet table. In compliance with the IRB protocol, and to keep the study as unobtrusive as possible, no individual weights or measures, demographic data, or historical behavioral data were collected. To collect food selection data, a researcher stood approximately fifteen to twenty feet from the end of either line in an obscure location, and for each individual, recorded a 1 when a food item was selected and a zero otherwise. Past experience with the binary coding of selected items on buffets has generated coding reliability of well over .90 ([13]), and it has been found that after a couple dozen observations, errors become rare. With this in mind, it was determined that multiple coders would not be needed unless sudden surges on the two buffet lines made this necessary. With this in mind, coders who were blind to the hypotheses were trained prior to breakfast and one was assigned to each of the two tables. A third coder, one of the researchers, helped with overflow coding and also dual-coded a subset of observations for a reliability check (coefficient alpha = .96 across the foods taken). Halfway through breakfast, the two coders switched tables to further avoid coder-specific bias. To maintain a high degree of reliability and to keep pace with the buffet goers, no attempt was made to measure how much of each item had been taken or how much room was left on their plate as they reached the end of the buffet.

Frequency tests using the Pearson Chi-square and Stuart-Maxwell tests were used to analyze whether the difference in serving lines had any impact on behavior. Following this, non-linear estimation techniques using the logistic density function examined the difference in the behavior of individuals between the two lines. Finally, pair-wise correlation coefficients and logistic regressions were calculated to understand whether foods were considered as substitutes or complements to each other.

## Results

**Figure 1:**
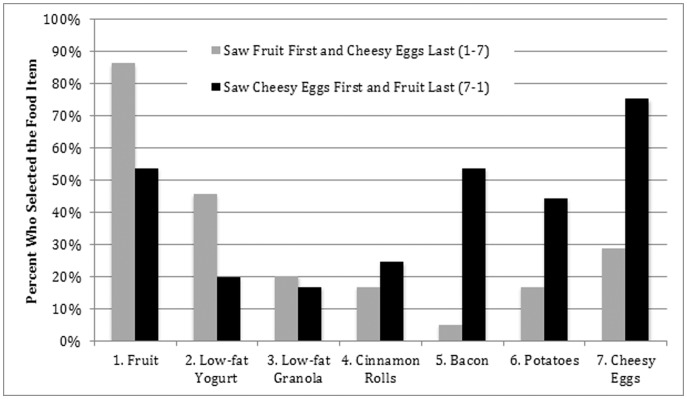
Food Presentation Order Influences the Percentage of Diners Who Selected Healthy or Unhealthy Foods. The percentages in this table are predicted percentages of individuals selecting an item in one of two buffet lines. These percentages were generated from a non-linear estimation procedure using the logistic density function.

As Figure 1 (Table 1) illustrates, and as all tests indicate, the order or sequence that food was presented to people biased what foods they selected. That is, a Pearson chi-square test statistic of 25.1 (p<0.001; 6 degrees of freedom) rejects the null hypothesis that food choice was independent of whether cheesy eggs or fruit were served first. An underlying assumption of this test statistic, however, is that the food choices people made were independent of one another. For example, selecting cheesy eggs was assumed to have no impact on whether bacon was chosen. Since this is clearly not a valid assumption, the Stuart-Maxwell test, which relaxes this independence assumption, was used to estimate whether the serving line affected food choice. This test generated a test statistic of 171.2 (p<0.001; 6 degrees of freedom), confirming the result from the previous test and indicating a strong interdependence between food choices, based on the line.

**Table 1:** Buffet Diners Tend to Select Whatever Foods They See First.

	Presented with Fruit Firstand Cheesy Eggs Last(1–7)	Presented with Cheesy EggsFirst and Fruit Last(7-1)	Odds Ratio	Z-Statistic	P-Value
1. Fruit	86.4%	53.8%	0.18	−3.74	<0.001
2. Low-fat Yogurt	45.8%	20.0%	0.30	−3.00	0.003
3. Low-fat Granola	20.3%	16.9%	0.80	−0.49	0.625
4. Cinnamon Rolls	16.9%	24.6%	1.60	1.04	0.297
5. Bacon	5.1%	53.8%	21.8	4.79	<0.001
6. Potatoes	16.9%	44.6%	3.95	3.21	0.001
7. Cheesy Eggs	28.8%	75.4%	7.57	4.97	<0.001

The percentages in this table are predicted percentages of individuals selecting an item in one of two buffet lines. These percentages were generated from a non-linear estimation procedure using the logistic density function.

Figure 1 shows that the first foods a person encountered in the buffet line were much more likely to be chosen than the last foods they encountered. Furthermore, there was a greater chance that diners in this study took the first option on the line (Table 1) – regardless of whether it was healthy (fresh cut fruit) or less healthy (cheesy eggs). Specifically, 86.4% of diners took fruit when it was offered first, compared to 53.8% of diners who took fruit when it was offered last (p<0.001). Similarly, 75.4% of all diners took cheesy eggs when they were offered first, while only 28.8% took cheesy eggs (p<0.001) when they were offered last. In total, the first three food items a person encountered in the buffet comprised about 65.7% of their total plate, regardless of whether those first items were cheesy eggs, fried potatoes, and bacon (55.6%) or whether they were fruit, low-fat yogurt, and low-fat granola (76.6%).

This difference in food selection by line also persisted in the fruit-first line for low-fat yogurt, and it persisted in the cheesy eggs-first line for both bacon and potatoes. In the fruit-first line, just under half of the diners took low-fat yogurt (45.8%), though diners in this line had over 3 times the odds of selecting low-fat yogurt when compared to diners in the other line (p = 0.003). Moreover, when cheesy eggs were served first, 44.6% of diners took potatoes and 53.8% took bacon, yet 64.6% took one or the other. On the other hand, less than 20% of the diners in the fruit first line took potatoes or bacon (16.9% and 5.1%, respectively). Thus, compared to diners in the fruit first line, diners passing through the line offering cheesy eggs first had nearly four times the odds (p = 0.001) of choosing potatoes and over 21 times the odds (p<0.001) of taking bacon.

While the presentation order of buffet foods triggered diners to take the items they encountered first, there was also evidence of a trigger effect ([12]), specifically in the cheesy eggs-first line. Pair-wise correlation coefficients in Table 2 suggest that placing less healthy foods first–cheesy eggs– seemed to trigger the selection of the next two calorically dense and highly palatable foods. When cheesy eggs were placed first, the correlation between choosing cheesy eggs and potatoes was 0.37 (p<0.01), the correlation between choosing eggs and bacon was 0.47 (p<0.001), and the correlation between choosing bacon and potatoes was 0.40 (p<0.01), indicating the strong complementarity between these foods.

**Table 2:** When Less Healthy Foods Are Offered First, They Trigger Other Unhealthy Choices.

	Potatoes	Bacon	Cinnamon Rolls	Low-fat Granola	Low-fat Yogurt	Fruit
**Cheesy Eggs**	0.37**	0.47***	−0.09	−0.22	−0.34**	0.04
**Potatoes**		0.40**	−0.15	−0.16	−0.22	−0.10
**Bacon**			−0.04	−0.16	−0.23	0.01
**Cinnamon Rolls**				−0.26*	−0.29*	−0.04
**Low-fat Granola**					0.70***	−0.16
**Low-fat Yogurt**						−0.08

The values in this table are pair-wise correlation coefficients between foods selected by diners in the line that served cheesy eggs first.

* p<0.05,

** p<0.01,

*** p<0.001.

In contrast, when fruit was served first, the only statistically significant correlations existed between selecting bacon (0.31; p<0.05) and fruit and cheesy eggs (−0.29; p<0.05), indicating potential substitutions between fruit and cheesy eggs. This suggests that placing the fruit first does not trigger the same demand response for calorically dense foods. Smaller, insignificant, correlations between the lower calorie options in both lines suggest there are greater variations in people’s preferences for fruit, low-fat yogurt, and low-fat granola (Table 3).

**Table 3:** When Healthy Foods Are Offered First, They Discourage Diners From Selecting Combinations of Less Healthy Foods.

	Low-fat Yogurt	Low-fat Granola	Cinnamon Rolls	Bacon	Potatoes	Cheesy Eggs
**Fruit**	−0.23	−0.05	−0.22	0.09	−0.09	−0.29*
**Low-fat Yogurt**		0.13	0.04	0.10	−0.05	0.09
**Low-fat Granola**			0.11	0.07	0.00	−0.04
**Cinnamon Rolls**				0.1	0.04	0.21
**Bacon**					0.31*	−0.15
**Potatoes**						0.21

The values in this table are pair-wise correlation coefficients between foods selected by diners in the line that served fruit first.

* p<0.05,

** p<0.01,

*** p<0.001.

In general, people tend to fill their plates with the foods offered first. Indeed, in this specific case, more than 75% of diners in either line took the first foods offered (Table 4). Interestingly, however, when less healthy foods were offered first, individuals took more total food items, potentially increasing the total calories on their plate. Specifically, in the cheesy eggs-first line, 78.5% of diners either took cheesy eggs, potatoes, or bacon (Table 4). Of these diners, 66.2% also served themselves fruit, low-fat yogurt, or low-fat granola. In contrast, when fruit was offered first, 96.6% either took fruit, low-fat yogurt, or low-fat granola, yet only 39% of these individuals took cheesy eggs, potatoes, or bacon. In other words, when cheesy eggs were first in line, and when diners had taken one of the first three foods, they also had more than three times the odds (p = 0.004) of taking one of the last three items. In summary, diners took 31% more items (2.20 to 2.89; p = 0.001) when cheesy eggs were served first.

**Table 4:** When Less Healthy Foods Are Served First, Diners Take More Total Foods.

	Presented withFruit First andCheesy Eggs Last(1–7)	Presented withCheesy EggsFirst and FruitLast (7-1)	Odds Ratio	Z-statistic	P-value
Percent who selected fruit *or* low-fat yogurt *or* low-fat granola	96.60%	66.20%	14.6	3.5	<0.001
Percent who selected fruit *and* low-fat yogurt or low-fat granola	44.10%	10.80%	6.53	3.92	<0.001
Percent who selected cheesy eggs *or* potatoes *or* bacon	39.00%	78.50%	5.7	4.32	<0.001
Percent who selected cheesy eggs *and* potatoes or bacon	8.40%	61.50%	17.28	5.35	<0.001

The percentages in this table are predicted percentages of individuals selecting an item in one of two buffet lines. These percentages were generated from a non-linear estimation procedure using the logistic density function.

## Discussion

In this study, two breakfast buffet lines served an array of seven different foods but arranged them in opposite orders – healthiest to least healthy versus least healthy to healthiest. Because food decisions are influenced by many factors ([11], [12], [14]), food order dramatically biases what diners take in two ways. First, a high percentage of diners took the first available food – over 75% of the diners in this study took the first food offered, and the first three foods a person encountered comprised 66% of all the foods they served themselves. This is an excellent illustration of the “first foods most” principle. Second, the first food they took also biased subsequent choices because they also took complementary foods – especially those that are calorically indulgent and palatable ([15], [16]).

Diners who passed through the cheesy eggs-first line tended to take similar side items that traditionally compliment eggs – bacon and fried potatoes. Yet, diners who served themselves in the fruit-first line demonstrated relatively little commonality in the other items they selected. Culturally, eggs, bacon, and potatoes (usually chunked and fried or grated and fried) are common breakfast foods that are served together. When cheesy eggs are served first, the diner may be obeying the cultural script [17] that bacon or potatoes should follow, so seeing and selecting eggs first triggers those subsequent choices. In addition, they also choose more total food items, potentially increasing the amount of calories they eat at a meal. Interestingly, although fruit is commonly served at breakfast, it is generally not served with any one specific complimentary food. Therefore, when fruit is served first, diners do not enter into any specific behavioral script (and are also less likely to choose eggs, bacon, and potatoes). Thus, fruit may act as a healthy trigger that disrupts the scripted behavior evident when cheesy eggs were served first.

While the results from this study are compelling, there are limitations that must be noted. First, while the results show a strong effect of food order on food choice, there is no evidence of what individuals actually consumed. Consumption measures would reveal what foods are actually eaten once diners return to their table. Second, portion sizes were not measured, thus we cannot determine if individuals who piled their plates high with foods at the beginning of the line would have had less room for foods further down the line. Third, no demographic information or food preference information was collected. Last, this study was conducted at a professional conference, and the results may not be generalizable to a broader population, such as with children in a school lunchroom who have less choice flexibility. A similar study conducted with a more heterogeneous population could speak to the generalizability of this finding.

This study compared two objective orderings of the foods served in a breakfast buffet. A more complete design would have used many more combinations. Additionally, in this study the relatively healthier foods were colder, and the less healthy foods were served hot. Preferences for a hot or cold breakfast may have led to food choice, and not necessarily the specific food offered. In addition, the smells from the hot foods may have increased the salience of the food, thus leading to the greater chance that individuals would take it ([18], [19]). These are areas to be addressed in future research.

Through principles of choice architecture and behavioral economics, one can leverage the “first foods most” behavior, as evident in this study, by putting healthier foods first. This easy, low-cost change to buffet lines can effectively help people eat better. Serving healthier foods first can be done by any conference hotel, Chinese buffet, catering company, school cafeteria, or even household to help both adults and children eat better [4]. Regardless of the location, there will likely be a similar impact on choice because of the impact that environments generally have on consumer choice ([20], [21]). Adjusting food arrangements is also a win-win strategy by not only nudging [20] consumers to eat better, but also by promoting healthier foods, helping consumers become slim by design.
